# ARDD 2020: from aging mechanisms to interventions

**DOI:** 10.18632/aging.202454

**Published:** 2020-12-30

**Authors:** Garik V. Mkrtchyan, Kotb Abdelmohsen, Pénélope Andreux, Ieva Bagdonaite, Nir Barzilai, Søren Brunak, Filipe Cabreiro, Rafael de Cabo, Judith Campisi, Ana Maria Cuervo, Marco Demaria, Collin Y. Ewald, Evandro Fei Fang, Richard Faragher, Luigi Ferrucci, Adam Freund, Carlos G. Silva-García, Anastasia Georgievskaya, Vadim N. Gladyshev, David J. Glass, Vera Gorbunova, Aubrey de Grey, Wei-Wu He, Jan Hoeijmakers, Eva Hoffmann, Steve Horvath, Riekelt H. Houtkooper, Majken K. Jensen, Martin Borch Jensen, Alice Kane, Moustapha Kassem, Peter de Keizer, Brian Kennedy, Gerard Karsenty, Dudley W. Lamming, Kai-Fu Lee, Nanna MacAulay, Polina Mamoshina, Jim Mellon, Marte Molenaars, Alexey Moskalev, Andreas Mund, Laura Niedernhofer, Brenna Osborne, Heidi H. Pak, Andrey Parkhitko, Nuno Raimundo, Thomas A. Rando, Lene Juel Rasmussen, Carolina Reis, Christian G. Riedel, Anais Franco-Romero, Björn Schumacher, David A. Sinclair, Yousin Suh, Pam R. Taub, Debra Toiber, Jonas T. Treebak, Dario Riccardo Valenzano, Eric Verdin, Jan Vijg, Sergey Young, Lei Zhang, Daniela Bakula, Alex Zhavoronkov, Morten Scheibye-Knudsen

**Affiliations:** 1 Center for Healthy Aging, Department of Cellular and Molecular Medicine, University of Copenhagen, Copenhagen, Denmark; 2 Laboratory of Genetics and Genomics, National Institute on Aging Intramural Research Program, National Institutes of Health, Baltimore, MD 21224, USA; 3 Amazentis SA, EPFL Innovation Park, Bâtiment C, Lausanne, Switzerland; 4 Center for Glycomics, Department of Cellular and Molecular Medicine, University of Copenhagen, Copenhagen, Denmark; 5 Department of Genetics, Albert Einstein College of Medicine, Bronx, NY 10461, USA; 6 Institute for Aging Research, Department of Medicine, Albert Einstein College of Medicine, Bronx, NY 10461, USA; 7 Novo Nordisk Foundation Center for Protein Research, University of Copenhagen, Copenhagen, Denmark; 8 Institute of Clinical Sciences, Imperial College London, Hammersmith Hospital Campus, London, W12 0NN, UK; 9 Experimental Gerontology Section, Translational Gerontology Branch, National Institute on Aging, National Institutes of Health, Baltimore, MD 21224, USA; 10 Buck Institute for Research on Aging, Novato, CA 94945, USA; 11 Department of Developmental and Molecular Biology, Institute for Aging Studies, Albert Einstein College of Medicine, Bronx, NY 10461, USA; 12 European Research Institute for the Biology of Ageing, University Medical Center Groningen, University of Groningen, The Netherlands; 13 Institute of Translational Medicine, Department of Health Sciences and Technology, Swiss Federal Institute for Technology Zürich, Switzerland; 14 Department of Clinical Molecular Biology, University of Oslo and Akershus University Hospital, 1478 Lørenskog, Norway; 15 School of Pharmacy and Biomolecular Sciences, University of Brighton, Brighton, UK; 16 Longitudinal Studies Section, Translational Gerontology Branch, National Institute on Aging, National Institutes of Health, Baltimore, MD 21224, USA; 17 Calico Life Sciences, LLC, South San Francisco, CA 94080, USA; 18 Department of Molecular Metabolism, Harvard T. H. Chan School of Public Health, Boston, MA 02115, USA; 19 HautAI OÜ, Tallinn, Estonia; 20 Division of Genetics, Department of Medicine, Brigham and Women's Hospital, Harvard Medical School, Boston, MA 02115, USA; 21 Regeneron Pharmaceuticals, Inc. Tarrytown, NY 10591, USA; 22 Departments of Biology and Medicine, University of Rochester, Rochester, NY 14627, USA; 23 SENS Research Foundation, Mountain View, CA 94041, USA; 24 Human Longevity Inc., San Diego, CA 92121, USA; 25 Department of Genetics, Erasmus MC, University Medical Center Rotterdam, Rotterdam, The Netherlands; 26 DNRF Center for Chromosome Stability, Department of Cellular and Molecular Medicine, Faculty of Health Sciences, University of Copenhagen, Copenhagen, Denmark; 27 Human Genetics, David Geffen School of Medicine, University of California, Los Angeles, CA 90095, USA; 28 Laboratory Genetic Metabolic Diseases, Amsterdam UMC, University of Amsterdam, Amsterdam, The Netherlands; 29 Section of Epidemiology, Department of Public Health, University of Copenhagen, Copenhagen, Denmark; 30 Gordian Biotechnology, San Francisco, CA 94107, USA; 31 Blavatnik Institute, Department of Genetics, Paul F. Glenn Center for Biology of Aging Research at Harvard Medical School, Boston, MA 94107, USA; 32 Molecular Endocrinology Unit, Department of Endocrinology, University Hospital of Odense and University of Southern Denmark, Odense, Denmark; 33 Department of Molecular Cancer Research, Center for Molecular Medicine, Division of Biomedical Genetics, University Medical Center Utrecht, Utrecht University, Utrecht, The Netherlands; 34 Departments of Biochemistry and Physiology, Yong Loo Lin School of Medicine, National University Singapore, Singapore; 35 Centre for Healthy Ageing, National University Healthy System, Singapore; 36 Department of Genetics and Development, Columbia University Medical Center, New York, NY 10032, USA; 37 Department of Medicine, University of Wisconsin-Madison and William S. Middleton Memorial Veterans Hospital, Madison, WI 53792, USA; 38 Sinovation Ventures and Sinovation AI Institute, Beijing, China; 39 Department of Neuroscience, University of Copenhagen, Denmark; 40 Deep Longevity Inc., Hong Kong Science and Technology Park, Hong Kong; 41 Juvenescence Limited, Douglas, Isle of Man, UK; 42 Institute of Biology of FRC Komi Science Center of Ural Division of RAS, Syktyvkar, Russia; 43 Institute on the Biology of Aging and Metabolism, Department of Biochemistry, Molecular Biology and Biophysics, University of Minnesota, Minneapolis, MN 55455, USA; 44 University of Pittsburgh, Pittsburgh, PA 15260, USA; 45 Institute of Cellular Biochemistry, University Medical Center Goettingen, Goettingen, Germany; 46 Department of Neurology and Neurological Sciences and Paul F. Glenn Center for the Biology of Aging, Stanford University School of Medicine, Stanford, CA 94305, USA; 47 OneSkin Inc., San Francisco, CA 94107, USA; 48 Department of Biosciences and Nutrition, Karolinska Institute, Stockholm, Sweden; 49 Department of Biomedical Sciences, University of Padova, Italy; 50 Institute for Genome Stability in Ageing and Disease, Medical Faculty, University of Cologne, Cologne, Germany; 51 Department of Pharmacology, School of Medical Sciences, The University of New South Wales, Sydney, NSW, Australia; 52 Departments of Obstetrics and Gynecology, Genetics and Development, Columbia University, New York, NY 10027, USA; 53 Division of Cardiovascular Medicine, University of California, San Diego, CA 92093, USA; 54 Department of Life Sciences, Ben-Gurion University of the Negev, Beer Sheva, Israel; 55 Novo Nordisk Foundation Center for Basic Metabolic Research, University of Copenhagen, Copenhagen, Denmark; 56 Max Planck Institute for Biology of Ageing, Cologne, Germany; 57 Longevity Vision Fund, New York, NY 10022, USA; 58 Insilico Medicine, Hong Kong Science and Technology Park, Hong Kong

**Keywords:** aging, interventions, drug discovery, artificial intelligence

## Abstract

Aging is emerging as a druggable target with growing interest from academia, industry and investors. New technologies such as artificial intelligence and advanced screening techniques, as well as a strong influence from the industry sector may lead to novel discoveries to treat age-related diseases. The present review summarizes presentations from the 7^th^ Annual Aging Research and Drug Discovery (ARDD) meeting, held online on the 1^st^ to 4^th^ of September 2020. The meeting covered topics related to new methodologies to study aging, knowledge about basic mechanisms of longevity, latest interventional strategies to target the aging process as well as discussions about the impact of aging research on society and economy. More than 2000 participants and 65 speakers joined the meeting and we already look forward to an even larger meeting next year. Please mark your calendars for the 8^th^ ARDD meeting that is scheduled for the 31^st^ of August to 3^rd^ of September, 2021, at Columbia University, USA.

## INTRODUCTION

**Figure 1:**
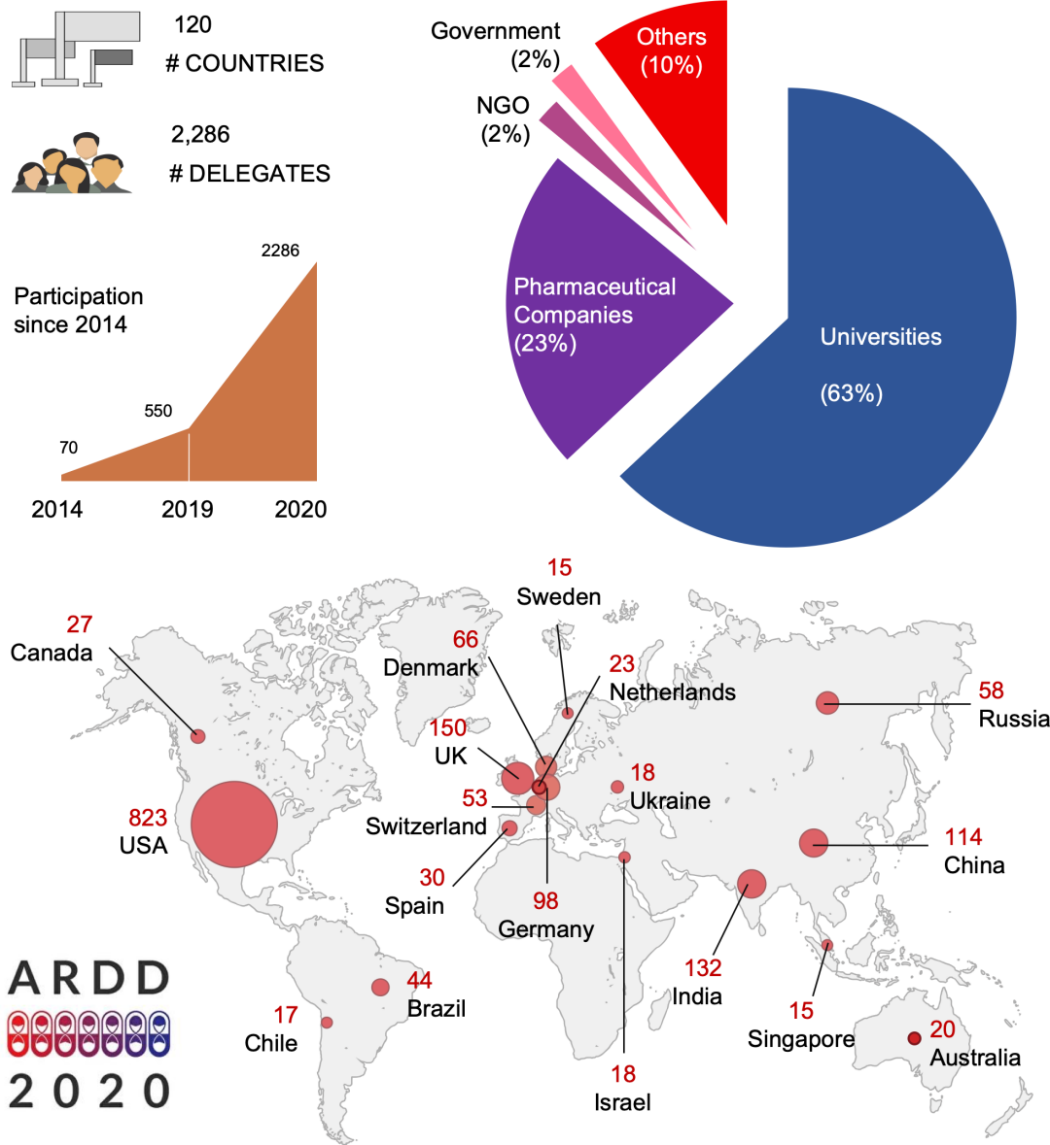
**Statistics from ARDD2020.**

A tremendous growth in the proportion of elderly people raises a range of challenges to societies worldwide. Healthy aging should therefore be a main priority for all countries across the globe. However, science behind the study of age-associated diseases is increasing and common molecular mechanisms that could be used to dissect longevity pathways and develop safe and effective interventions for aging are being explored. In this regard, novel methodologies using the power of artificial intelligence (AI) are emerging to cope with the massive amount of data that are becoming available [1]. A collaborative effort based on transfer of technology and knowledge between academia and industry is also needed to accelerate aging discoveries and facilitate better transition of effective interventions into clinics. To accelerate this, the Aging Research and Drug Discovery (ARDD) meeting was founded seven years ago in Basel, Switzerland. This year’s meeting, organized by Alex Zhavoronkov, Insilico Medicine, Morten Scheibye-Knudsen, University of Copenhagen and Daniela Bakula, University of Copenhagen, was particularly challenging due to the ongoing COVID-19 pandemic. The 7^th^ ARDD meeting, 1^st^ to 4^th^ of September 2020, moved online with local hosting at the University of Copenhagen. We were very fortunate to have 65 fantastic speakers and more than 2200 ‘ARDDists’ (Figure 1). This report provides an overview of the presentations covering topics on some of the latest methodologies to study aging, molecular characterization of longevity pathways, existing aging interventions and the importance of aging research for the global society and economy.

## Novel approaches to study aging

The progress in discoveries of basic mechanisms of aging as well as development of novel interventional strategies depends primarily on approaches and tools that are used in the lab. During the last couple of decades, methodological strategies for emerging big data from various cell-, tissue- and organ-types have accelerated towards development of high-throughput screening techniques and computational approaches using the power of artificial intelligence [1]. However, despite the capacity of data analysis, existing methods are constantly improving and becoming integrated as a part of intervention-screening platforms. Martin Borch Jensen, Gordian Biotechnology, San Francisco, USA, presented their approach for conducting high-throughput screens of many therapies in a single animal, using single-cell sequencing. By identifying cells within a diseased tissue that appear healthy after receiving one of many interventions, they are able to test *in vivo* efficacy of therapies much faster than traditional drug development. Another achievement in the development of advanced technology has been made in the area of proteomics, discussed by Andreas Mund, University of Copenhagen, Denmark. In particular, single cell proteomes can be analysed to extract valuable information regarding disease mechanisms. Its integration with multiplexed imaging of human-derived tissue samples and deep learning techniques enables the creation of an advanced deep visual proteomics pipeline for discovery of novel biomarkers and effective therapeutics that could potentially be used in clinics [2]. Further, Ieva Bagdonaite, University of Copenhagen, Denmark, underlined the value of investigating protein glycosylation that undergoes dynamic changes in age-associated diseases, as well as highlighted the importance of mapping complex glycoproteome using advanced quantitative proteomics for both developing efficient therapies and biomarker discovery [3].

Benefits of omics-based big data analysis were also presented by Christian Riedel, Karolinska Institutet, Sweden, who developed an advanced screening approach for geroprotector discovery. In this approach, screening of novel compounds is based on the transcriptome analysis of human tissues from people of varying ages and the application of machine learning to create age classifiers that predict biological age [4]. He looks for compounds that can shift transcriptomes of “older” towards “younger” tissues and thereby identifies drug candidates with potential geroprotective capabilities. Transcriptomics and other omics-based tools are also actively used by Vadim Gladyshev’s team, Brigham and Women’s Hospital, Harvard Medical School, USA. Particularly, comparative analysis of global transcriptomics and metabolomics data across species with different lifespan, across known longevity interventions, and across cell types with different lifespan can be used to develop an unbiased approach for the discovery of novel longevity interventions [5, 6]. Such approach can be intensely applied to determine the relationship among different types of interventions and its association with lifespan extension [7]. Identification of common signatures of longevity also raises great opportunities for high-throughput screening for novel compounds that are candidates for lifespan extension. Besides interventions, genetic studies enable the identification of novel factors contributing to aging. Using available genotyping data and genetic traits from two human cohort studies, ultra-rare damaging mutations were identified including rarest protein-truncating variants that negatively affect lifespan and healthspan [8]. Interestingly, genetic variation that supports longevity is also protective against COVID-19, which emerges as a disease of aging [9, 10]. A genetic approach to understand aging in humans was also described by Yousin Suh, Columbia University, USA. Studies of common and rare genetic variants in centenarians can be used to dissect longevity-associated genetic variants that may potentially be applied to understand the molecular basis of healthy aging as well as to develop new therapies for improving healthspan [11, 12].

Novel animal models are also being explored for the study of aging. Dario Riccardo Valenzano, Max Planck Institute for Biology of Ageing, Cologne, Germany, demonstrated how host-microbiota interactions can be important determinants for maintaining homeostasis and modulating lifespan using the short-lived model organism African turquoise killifish [13, 14]. He showed that fecal transplantation of young microbiota to old animals rescued age-dependent decrease in abundance of microbiota and extended the lifespan of the fish. Modulation of microbial function can also impact the effect of potential pharmacological interventions in hosts, as was highlighted by Filipe Cabreiro, Imperial College London, UK, who suggested that age-dependent changes in gut microbial composition can be potentially considered one of the hallmarks of aging [15]. Using the well-known pro-longevity drug metformin and a combination of model organisms such as *C. elegans* and *D. melanogaster* together with computational approaches for modelling human microbiome data, Filipe’s team showed that diet can impact the beneficial effect of metformin. His team developed a high-throughput screening platform to identify dietary metabolites and microbial molecular pathways that are responsible for the effect of metformin on health- and lifespan [16]. In worms, this and other potential aging interventions can be tested using a novel approach to measure health by atomic force microscopy based on the assessment of worm stiffness and cuticle senescence (roughness) across age spectrum [17].

## Deep aging clocks

Application of artificial intelligence for the development of novel therapeutics and biomarker discovery have been actively highlighted during the ARDD meeting. In particular, for the last couple of years several aging clocks have been developed to predict chronological and biological age based on certain clinical parameters [18]. Steve Horvath, University of California, Los Angeles, USA, demonstrated their importance in predicting not only chronological age, but also mortality risks across mammalian species referring to second generation epigenetic clocks, including PhenoAge and GrimAge [19, 20]. The translational value of epigenetic clocks was also highlighted in regard to testing aging interventions and their application in clinical trials [21]. The assessment of health state and life expectancy using deep learned clocks was further presented by Alice Kane, Harvard, USA. She developed a deep learned aging measure using a frailty index as a fast non-invasive mortality predictor for mice [22]. Another talk on estimation of chronological age was given by Anastasia Georgievskaya, Haut.AI, Tallinn, Estonia. Anastasia took an advantage of face and hand images from different age groups to create multimodal age prediction analyses as a part of a pipeline for the development of non-invasive visual biomarkers of human aging [23]. The importance of deep learning applications in healthcare and biomarker discovery was also discussed by Polina Mamoshina, Deep Longevity, Hong Kong. In particular, because different biological aging clocks may be associated with different aging processes, Deep Longevity aims to combine multiple clocks to estimate and monitor biological age over time [18, 24].

Interestingly, in humans, drug repurposing and its efficiency can be estimated not only by applying various aging clocks, but also using life-course trajectories and health-to-disease transition analyses presented by Søren Brunak from Novo Nordisk Foundation Center for Protein Research, University of Copenhagen, Denmark [25]. Using large datasets on millions of patients, this *in silico* approach enables the understanding of how one disease follows another and estimates if certain genes are linked to diseases [26]. The use of single patient disease trajectories spanning up to 20 years when predicting intensive care mortality highlighted how aging data and machine learning can be made actionable at the bedside as opposed to statistical assessment of larger groups of individuals [27].

## Genome maintenance in aging and longevity

Age-dependent alterations in cellular pathways lead to a decline in organ function and progression of disease. Understanding what causes aging and examining common patterns of changes on gene, protein and post-translational levels during the lifespan and across multiple tissues is one of the challenging tasks for aging researchers. Jan Vijg, Albert Einstein College of Medicine, USA, underlined that somatic mutations, including point mutations and genomic rearrangements, accumulate during aging and may contribute to mortality and disease [28]. Applying single-cell sequencing allows the identification of an age-dependent exponential increase in mutation frequency in human B lymphocytes [29] and liver hepatocytes [30], and propose mechanisms of how *de novo* mutations accumulate from early embryogenesis to adulthood and old age, and lead to the development of disease [28]. A topic of chromosomal aging was also highlighted in regard to reproductive lifespan by Eva Hoffmann, University of Copenhagen, Denmark. Investigations of changes in fertility rate during aging showed that chromosome errors and aberrations in oocytes control natural fertility in humans [31]. Based on current knowledge of genetic regulation of reproductive aging, interventions for pregnancy loss are being developed and were discussed during the ARDD meeting.

Interestingly, a genetic network that controls reproductive aging and somatic maintenance is primarily related to pathways associated with DNA repair and cell cycle regulation. Substantial research in aging has been done towards investigating nuclear DNA damage, which associates with multiple hallmarks of aging [32], as well as developing interventional strategies for protecting the aging genome [33]. Björn Schumacher, University of Cologne, Germany, characterized molecular consequences of DNA damage either in germline or somatic cells [34, 35] and examined molecular pathways that regulate somatic maintenance using *C. elegans* as a model organism. His recent discovery of epigenetic modifiers that are required for maintaining lifespan after DNA damage [36] shed light on novel molecular pathways linking DNA damage, epigenetics and longevity. A connection between DNA damage/repair, post-translational modifications and longevity was also presented by Vera Gorbunova, University of Rochester, USA, who applies a comparative biology approach to study short- and long-lived animals. Her team showed that the DNA double strand break (DSB) repair efficiency shows strong positive correlation with maximum lifespan across mammalian species. Higher DNA repair efficiency in long-lived species was, in large part, due to the higher activity of the histone deacetylase and mono-ADP-rybosylase, Sirtuin 6 (SIRT6) [37]. Vera’s talk also included unpublished data in humans showing that rare missense mutations in SIRT6 sequence identified in human centenarians are associated with more efficient DNA DSB repair. The functional role of SIRT6 in DNA repair also includes acting as a sensor of DSB, observed by Debra Toiber, Ben Gurion University of the Negev, Israel. Her data illustrates that SIRT6 directly binds to DNA, is recruited to the site of damage independently of PARP, MRE11 and KU80 and triggers activation of a DNA damage response [38]. Considering that SIRT6 depletion leads to accelerated aging and neurodegeneration phenotypes in mice, targeting it could be a potential strategy for the development of novel neuroprotective therapeutics [39, 40].

Importantly, genome maintenance and age-dependent changes in gene expression patterns are primarily dependent on chromatin state and epigenetic modifications [41]. David Sinclair, Harvard, USA, showed that DSBs may drive age-dependent epigenetic alterations and loss of cellular identity. Using a transgenic mouse system for inducible creation of DSBs, he revealed that loss of epigenetic structures, an accumulation of epigenetic noise and increased predicted DNA methylation changes increase with age and DNA damage [42, 43]. Importantly, the introduction of an engineered vector expressing Yamanaka transcription factors, excluding c-Myc, regenerated axons after optic nerve crush injury and restored vision in old mice [44]. The effect was dependent on the DNA demethylases Tet1, Tet2 and TDG and was accompanied by a reversal of methylation patterns and resetting the DNA methylation clock. Interestingly, epigenetic modifications in aging were studied also in other model organisms. In worms, one of the euchromatin-associated epigenetic marks known to affect lifespan is H3K4me3, controlled by the COMPASS complex [45]. Carlos Silva Garcia, Harvard, USA, showed that COMPASS-related longevity in *C. elegans* is dependent on activation of SREBP1, a master regulator of lipid metabolism leading to an increase in monosaturated fatty acids that is required for lifespan extension in COMPASS-deficient *C.elegans* [45], and presented novel data on the involvement of CREB-regulated transcriptional coactivator CRTC1 in COMPASS-mediated lifespan extension [46]. Here, Alexey Moskalev, the Russian Academy of Sciences, Russia, discussed mechanisms associated with lifespan-prolonging effects of chromatin modifier E(z) histone methyltransferases in *D. melanogaster*. The increase in lifespan of heterozygous E(z) mutant flies is associated with higher resistance to different stressors and changes in expression of genes related to immune response, cell cycle, and ribosome biogenesis [47].

## Longevity pathways

Longevity-associated molecular pathways are actively being explored in different model organisms. In rodents, differential gene expression in multiple tissues were described by David Glass from Regeneron, Inc., USA. Studies revealed an increase in gene expression variability during aging where bioenergetics pathways were identified to be significantly down-regulated in kidney, skeletal muscle and liver, while inflammatory signaling was upregulated in these tissues [48]. While old animals demonstrated increased activity of mammalian Target Of Rapamycin (mTOR) in skeletal muscle, it was highlighted that mTOR needs to be down-regulated for healthspan benefits but not completely inhibited. By using rapalogs, inhibitors of mTOR, the authors observed improvements in the kidney of old rats and identified key regulators, including c-Myc, that are involved in the beneficial effect of mTOR inhibition [49]. In a mouse study, partial mTOR down-regulation was also shown to be beneficial for skeletal muscle – decreasing degeneration/regeneration, and, surprisingly, increasing skeletal muscle mass [50]. Another mechanism by which down-regulation of mTOR signaling leads to longevity, was presented by Collin Ewald, ETH Zurich, Switzerland. Collin’s team discovered a hydrogen sulfide pathway as a potential longevity mechanism that is up-regulated by ATF4 in dietary restriction (DR) as a stress response to a decrease in global mRNA translation [51]. Interestingly, hydrogen sulfide has already been shown to possess beneficial effects in age-related diseases with currently running clinical trials for cardio-vascular improvements (NCT02899364 and NCT02278276). However, in addition to activation of hydrogen sulfide signaling, decreased mRNA translation and ATF4 expression have a strong regulatory link to the mitochondrial translation machinery that was also shown to impact longevity [52]. Riekelt Houtkooper, Amsterdam UMC, Netherlands showed that down-regulation of mitochondrial ribosomal proteins extends lifespan [52] and presented novel data that disruption of mitochondrial dynamics [53] synergizes with reduced mitochondrial mRNA translation in *C.elegans* [54]. Marte Molenaars, Amsterdam UMC, Netherlands demonstrated that there is a balance between mRNA translation in the cytosol and in mitochondria, and inhibiting mitochondrial translation leads to the repression of cytosolic translation and lifespan extension via *atf-5*/ATF4 [55].

Another molecular mechanism tightly related with mitochondria and other organelles maintenance that is impaired during aging is autophagy [56]. Ana Maria Cuervo, Albert Einstein College of Medicine, USA, presented the importance of selective, and in particular, chaperone-mediated autophagy (CMA) in aging and age-related diseases [57]. Her recently developed mouse model to monitor CMA *in vivo* revealed that CMA activity is activated upon starvation in multiple organs, but that there are cell-type specific differences in this response [58]. CMA decline in most organs and tissues at different rate and contributes to loss of proteostasis and subsequent cell function. In the case of neurons, reduced CMA in mice leads to gradual alterations in motor-coordination and cognitive function suggesting that targeting selective autophagy can be a treatment option in neurodegenerative diseases [59]. Anais Franco Romero, University of Padova, Italy, presented data on the identification of novel FOXO-dependent genes that are related to longevity. One of the hits, the MYTHO gene, was found to be highly up-regulated in old mice and humans compared to young ones, and is associated with impairments in the autophagy machinery and motor function alterations in *C. elegans* and *D. Rerio* model organisms. A novel mechanism linking lysosomal function and mitochondria was presented by Nuno Raimundo, Universitätsmedizin Göttingen, Germany, who studied the consequences of impaired lysosomal acidification in aging and the development of neurodegeneration. Specifically, the data illustrates that blockage of lysosomal acidification via vATPase inhibition leads to the accumulation of iron in lysosomes and cellular iron deficiency resulting in impaired mitochondrial function and development of inflammation both in cultured neurons and in the brain of mice [60].

Importantly, because multiple cellular maintenance pathways associated with longevity requires high energy consumption, mitochondrial function can be considered to possess a pivotal role to affect biological aging. Luigi Ferrucci, National Institute on Aging - NIH, USA, highlighted that restoration of mitochondrial biogenesis and function may be achieved by temporary but not long-term blockage of major energy-consuming regulatory pathways. More specifically, Luigi’s talk was aimed at explaining why mitochondrial function declines with age and presented evidence of reduced resting muscle perfusion, altered lipid biosynthetic pathways and impaired activity of the carnitine shuttle [61, 62].

## Lifestyle strategies for metabolic interventions

Identification of longevity-associated molecular pathways and the discovery of novel biomarkers of human aging goes along with the development of effective strategies for interventions. Currently, multiple interventions have been developed to target cellular metabolism, which is considered to possess a critical function in aging process [63]. Lifestyle interventions, including diet and its nutrient composition, regulate metabolic balance and affect lifespan across different species. Andrey Parkhitko, University of Pittsburgh, USA, discussed the role of the non-essential amino acid tyrosine in aging. In fly experiments, tyrosine levels decrease with age, accompanied by an increase of tyrosine-catabolic pathways. Preliminary data revealed that down-regulation of enzymes of tyrosine degradation, including the rate-limiting tyrosine aminotransferase (TAT), extends the lifespan of *D. Melanogaster* [64]. How protein affects lifespan has been further explored by Dudley Lamming, University of Wisconsin, USA [65]. Dudley’s team dissected the role of essential dietary amino acids in regulating lifespan. Reduction of branched chain amino acids (Leucine, Isoleucine and Valine) increases metabolic health, reducing adiposity and improving glucose tolerance in mice [66]. Further studies revealed that the low BCAA diet possess geroprotective properties, extending the lifespan of two progeroid mouse models, improving metabolic health in wild-type mice throughout their lifespan, and extending the lifespan and reducing the frailty of wild-type male, but not female, mice. These effects may be mediated in part by a sex-specific effect of a low BCAA diet on mTORC1 signaling [67]. Furthermore, Heidi Pak, University of Wisconsin, USA, illustrated that fasting is required for the beneficial effect of caloric restriction on healthspan and lifespan (unpublished data). Specifically, fasting was necessary to detect improvements in insulin sensitivity and to obtain the distinct metabolomic and transcriptomic signatures observed in caloric restricted male mice. Similarly, Pam Taub, UC San Diego, USA described the beneficial role of fasting as a part of lifestyle strategies for patients with cardiometabolic disease [68]. Importantly, the circadian rhythm was highlighted to have an important role for driving metabolism and affecting the efficiency of interventions. One dietary intervention that does not affect the robustness of the circadian rhythm is time-restricted eating (TRE). Pam’s recently published study illustrated that 10 h TRE can be used as a safe and effective lifestyle intervention, together with standard medications that are applied for treatment of cardiometabolic syndrome. However, besides metabolic diseases, fasting and caloric restriction display beneficial effects also in diseases associated with premature aging. Jan Hoeijmakers, Erasmus Medical Center Rotterdam, Netherlands, presented data that caloric restriction positively affects behavior and extends the lifespan in ERCC1-deficient progeroid mice, and reduces tremors and improves the cognitive function in a human patient.

Another lifestyle intervention that has a beneficial role for healthy aging is exercise. Thomas Rando, Stanford, USA, underlined the regenerative potential of skeletal muscles from young species that decline during aging. He showed that exercise in the form of running improves functionality of muscle stem cells almost to the level of young cells and increases the aged muscle capacity to repair injury in mice [69]. The improved function of muscle stem cells in old animals after exercise was associated with up-regulation of Cyclin D1, suppression of TGFbeta signaling and an exit from quiescence [69]. However, besides a decreased capacity of muscle regeneration, a decline in muscle function is also known to occur during aging. Gerard Karsenty, Columbia University, USA highlighted that age-dependent decline in muscle function and exercise capacity can be restored using osteocalcin. Circulating levels of this bone-derived hormone dramatically decreases already in middle age, surges after running and this hormone favors muscle function during exercise without affecting muscle mass, through two mechanisms in part. First, osteocalcin signaling in myofibers promotes uptake of glucose and fatty acids and the catabolism of these nutrients to produce ATP molecules needed for muscle function during exercise. Second, osteocalcin signaling in myofibers up-regulates the release in the circulation of muscle-derived interleukin-6 that in a feed forward loop increases the release of osteocalcin by bone during exercise and thereby exercise capacity [70]. Injection of osteocalcin increases the exercise capacity, fully restores muscle function and increases muscle mass in aged mice [70, 71]. Recent data also revealed that osteocalcin outperforms one of the leading compounds that is being tested for sarcopenia already in late clinical trials.

Benefits of exercise training for muscle function were also described in the context of maintenance of nicotinamide adenine dinucleotide (NAD^+^) metabolism by Jonas Thue Treebak, University of Copenhagen, Denmark. A rate-limiting enzyme of NAD^+^ metabolism, nicotinamide phosphoribosyltransferase (NAMPT), declines with age and was shown to be the only enzyme from the NAD^+^ salvage pathway that is restored by aerobic and resistance exercise training in human skeletal muscle [72]. Recent studies revealed that knockout of NAMPT in mouse skeletal muscle leads to a strong reduction in muscle function, dystrophy and premature death, suggesting a crucial role of NAMPT for maintaining NAD^+^ levels in skeletal muscle.

## Pharmacological approaches to modulate healthspan and lifespan

Molecular and therapeutic importance of NAD^+^ metabolism for aging was underlined in multiple talks at the ARDD meeting. Eric Verdin, Buck Institute, USA introduced the concept of competition among major NAD^+^-utilizing enzymes for NAD^+^ that may explain its age-dependent decline across multiple tissues [73]. The main focus of the talk was CD38, a NAD^+^-metabolizing enzyme that increases with age in adipose tissues [74]. Verdin’s team discovered that CD38 activity is increased in M1 macrophages during aging and its activation depended on key cytokines from the senescence-associated secretory phenotype (SASP) secreted by senescent cells. Brenna Osborne, University of Copenhagen, Denmark further illustrated that depletion of CD38 appears to exacerbate some of the aging phenotypes in the mouse model of Cockayne syndrome, where another major NAD^+^-utilizing enzyme poly(ADP) ribose polymerase 1 (PARP1), is hyperactivated. Overall, current data suggest that a crosstalk between NAD^+^-utilizing enzymes needs to be continuously investigated in order to develop safe and effective interventions targeting NAD^+^ metabolism. However, precursors of NAD^+^ are actively being tested in various age-associated disorders. Evandro Fei Fang, University of Oslo, Norway underlined the importance of the NAD^+^-mitophagy/autophagy axis in aging and neurodegeneration and presented data on how impairment of this axis contributes to the progression in accelerated aging diseases as well as in the most common dementia, the age-predisposed Alzheimer’s disease [75, 76]. Induction of mitophagy either by NAD^+^ or other mitophagy stimulators inhibits amyloid-beta and p-Tau aggregates, as well as improves memory impairments in several models of Alzheimer’s disease [77, 78]. Similar results were observed by Lene Juel Rasmussen from Center for Healthy Aging, University of Copenhagen, Denmark. Lene’s team uses *in vitro* and *in vivo* animal models with a deficiency in the DNA repair gene REV1, which causes replication stress and premature aging. Suppression of REV1 is associated with high PARP1 activity, low endogenous NAD^+^ and low SIRT1 expression [79]. Presented data showed that mitochondrial dysfunction and morphology changes were suppressed, and autophagy was increased after nicotinamide riboside (NR) supplementation in REV1-deficient cells and that NR increased the lifespan and healthspan of REV1-deficient nematodes. Importantly, the underlying cause of the development of premature aging disorders described before are impairments in genes associated with DNA repair [80]. Morten Scheibye-Knudsen, University of Copenhagen, Denmark, demonstrated the importance of targeting DNA repair for healthy aging and illustrated how the power of AI can be applied to find novel DNA repair stimulators. Particularly, an *in silico* approach enabled the identification of novel compounds that are able to delay replicative aging and reverse senescent phenotypes in multiple primary cells, as well as improve the behavior and extend the lifespan in wild-type *D. Melanogaster* (unpublished data).

Another recently uncovered molecule that is able to improve mitochondrial function via mitophagy is Urolithin A, a gut microbiome metabolite known to improve mitochondrial function via mitophagy, increases muscle function and possesses geroprotective features across multiple species [81]. Pénélope Andreux, Amazentis, Switzerland presented results from a double blinded placebo controlled study showing that urolithin A administration in healthy elderly people is safe and was bioavailable after single or multiple doses over a 4-week period [82]. Oral consumption of urolithin A decreased plasma acylcarnitines, a sign of improved systemic mitochondrial function, and displayed transcriptomic signatures of improved mitochondrial and cellular health in muscle. Interventions targeting autophagy pathways were also highlighted by Rafael de Cabo, National Institute on Aging-NIH, USA, in the context of obesity and metabolic health. Recent data showed that disulfiram treatment prevents high-fat diet-induced obesity in mice by reducing feeding efficiency, decreasing body weight, and increasing energy expenditure [83]. Moreover, disulfiram prevents pancreatic islet hyperplasia and protects against high-fat diet-induced hepatic steatosis and fibrosis. Further experiments uncovered common molecular signatures after disulfiram treatment, revealing pathways associated with lipid and energy metabolism, redox, and detoxification and identified autophagy as one of the key targets by which disulfiram mediates its beneficial effects in cell culture [84]. The link between metabolic health and age-related bone loss was highlighted by Moustapha Kassem, Molecular Endocrinology Unit, University of Southern Denmark, Denmark, who suggested targeting skeletal mesenchymal stem cells (MSC) for the treatment of age-related osteoporosis. A decline in bone marrow composition, as well as alterations in the function of MSC in bone remodelling, are known to occur during aging [85]. The Kassem team identified the KIAA1199 protein to be highly secreted from hMSCs during osteoblast differentiation *in vitro* [86] and is associated with recruitment of hMSC to bone formation sites [85].

Another “classical” pro-longevity pathway that is explored for the development of aging interventions is the IGF signaling pathway. For example, targeting IGFBP-specific PAPP-A protease using genetically modified mouse models leads to lifespan extension [87, 88]. Here, Adam Freund, Calico Life Sciences LLC, USA, investigated targeting PAPP-A using antibodies. RNA sequencing revealed treatment with anti-PAPP-A to down-regulate collagen and extracellular matrix genes across multiple tissues. Further investigations identified MSCs to be a primary responder to PAPP-A inhibition. Restraining MSC activity is likely to be a mechanism driving a systemic response of tissues to PAPP-A inhibition. However, further experiments are required for the development of safe and effective therapeutic strategies for reducing IGF signaling.

Importantly, IGF-1 and other pro-aging factors may trigger activation of the NF-kB signaling cascade leading to inflammation and the development of senescent phenotypes, suggesting that NF-kB plays a key role in modulating the aging process [89, 90]. Lei Zhang, University of Minnesota, USA, applied an *in silico* approach to screen compounds capable of disrupting IKKβ and NEMO association thereby inhibiting NF-kB transcriptional activation [91]. A small molecule called SR12343 was identified to suppress lipopolysaccharide (LPS)-induced acute pulmonary inflammation in mice and attenuate necrosis and muscle degeneration in a mouse model of Duchenne muscular dystrophy [91]. SR12343 also attenuated senescent cell phenotypes *in vitro* as well as in mouse models of premature aging. A late life intervention with SR12343 in naturally aged mice demonstrated a decrease in senescent markers in liver and muscle. Hence, pharmacological targeting of NF-kB activation offers considerable potential for improving healthspan.

## Interventions targeting senescent cells

Notably, studies of multiple interventions in different aging models include examinations of various markers of cellular senescence. Its significance for the aging process has been shown multiple times across model systems [92]. Senescent cells occur in all organs, including post-mitotic brain tissues, during aging and at sites of age-related pathologies. The SASPs of senescent cells lead to chronic inflammation and may contribute to the development of various cellular phenotypes associated with aging and diseases. Hence, a novel class of drugs targeting senescent cells are emerging, including senolytics (selective elimination of senescent cells) and senomorphics (selective modification of senescent cells). However, it should be considered that cellular senescence is a balancing act between its beneficial and detrimental roles in maintaining tissue homeostasis, as described by Judith Campisi from Buck Institute, USA. For instance, removal of senescent cells by senolytic drugs is one strategy to combat aging phenotypes [93]. However, no single senolytic drug eliminates all senescent cells, likely due to the heterogeneity among cells and distinct cell-type specific differences and variations in the SASP [94]. Moreover, it was highlighted that the SASP also varies depending on the senescence inducer [93] underlining the question: “what drives cells into senescence during natural aging?”. In particular, this question was addressed by Kotb Abdelmohsen, National Institute on Aging - NIH, USA, who presented data on the identification of a transcriptome signature of cellular senescence based on RNA sequencing [95]. His team identified the microRNA miR-340-5p to be highly expressed in senescence triggered by several inducers across multiple cell types. MiR-340-5p promotes senescence through the downstream effector Lamin B receptor (LBR). They also discovered that miR-340-5p is senolytic-associated or *senomiR* that sensitizes senescent cells to senolytic drugs.

Several strategies were proposed to target senescent cells. Marco Demaria, ERIBA, Netherlands, demonstrated the important role of oxygen in the development of the senescence phenotype [96]. Data illustrated that growth arrest, lysosomal activity and DNA damage signalling were similarly activated in senescent cells cultured at 1% or 5% oxygen, but induction of the SASP was suppressed by low oxygen. Tissues exposed to low oxygen also expressed a lower SASP than more oxygenated ones. It was demonstrated that hypoxia restrains SASP via AMPK activation and mTOR inhibition, and that intermittent treatment with hypoxia mimetic compounds can serve as a potential strategy for the reduction of SASP *in vivo*. Further, Peter de Keizer, University Medical Center Utrecht, Netherlands underlined again the problem of the existence of distinct subtypes of cellular senescence and the absence of senescence-specific markers. A strategy of FOXO4-p53 targeting using a designed FOXO4 peptide and other FOXO4-p53 inhibitory compounds can be applied to selectively eliminate senescence cells that appear during aging, as well as “senescence-like” chemoresistant cancer cells [97]. Laura Niedernhofer, University of Minnesota, USA, demonstrated a senolytic activity of fisetin, a natural flavonoid that improves the health- and lifespan in mouse models of normal and accelerated aging [98]. It was highlighted that several clinical trials with fisetin, also in regard to COVID-19, are under way.

Another application of small molecules, resveralogues, to target senescent cells by reversing their phenotype was presented by Richard Faragher, University of Brighton, UK [99]. A range of compounds based on resveratrol were able to reverse senescent phenotypes and restore proliferative capacity by altering mRNA splicing and moderating splicing factor levels [100]. Those compounds that were also able to activate SIRT1 demonstrated greater abilities to rescue cells from the senescence state [101].

Interestingly, screening of novel molecules using advanced AI-based tools and targeting senescent cells is also emerging. Carolina Reis, OneSkin, USA, underlined the importance of skin aging and illustrated why targeting senescent cells with novel senotherapeutic compounds can promote skin health in order to delay the onset of age-related diseases. Cell-based drug screening identified a lead compound, OS-1, that was able to reduce senescent cell burden and protect cells from UVB-induced photoaging. In addition, OS-1 was shown to reduce the molecular age of the skin using their developed skin-specific epigenetic clock [102] and showed benefits for skin health in a clinical study. Further experiments are being performed to examine whether OS-1 affects lifespan and healthspan in model organisms.

Besides that application of lifestyle strategies that in many cases can mimic pharmacological therapies or possess synergetic effects for healthspan and lifespan, one should consider more upstream events on the level of prediction of disease. In this regard, development of non-invasive biomarkers for human aging acquires special significance. Majken Jensen, University of Copenhagen, Denmark illustrated the value of investigating high-density lipoprotein (HDL) in the context of cardiovascular diseases and demonstrated HDL containing apoC3 to be the only subtype of HDL that was associated with higher risk of heart disease [103]. The problem of missing stable biomarkers for dementia prediction and Alzheimer disease was also underlined in Jensen’s talk. Recently, published data revealed plasma apoE in HDL and lacking apoC3 was associated with lower dementia risk and better cognitive function [104]. This and other novel biomarkers for Alzheimer disease can be discovered using non-targeted proteomic profiling in cerebrospinal fluid (CSF) [105]. The importance of aging of the tissue producing the CSF was further demonstrated by Nanna MacAulay, University of Copenhagen, Denmark. Alterations associated with dysregulation of CSF can lead to several pathologies, including stroke-related brain edema and hydrocephalus. Water cotransporter mechanisms, rather than conventional osmotic driving forces, were highlighted to play a crucial role in the production of CSF and secretion from the blood to the brain. The Na^+^/K^+^/2Cl^-^ cotransporter (NKCC1) was identified to mediate approximately half of the CSF production, and thus provides opportunities for developing novel interventional strategies for pathologies associated with elevated brain fluid levels [106].

## Challenges in aging: science, society and economy

Currently, our understanding of the molecular basis of aging and age-associated diseases is improving. However, challenges in the aging field exist and refer to both science and society in general. Nir Barzilai, Albert Einstein College of Medicine, USA, highlighted several concerns including (1) the translational value of identified longevity mechanisms and effective interventions from animals to humans, (2) the discovery of reliable biomarkers for estimation of efficiency of various therapies and (3) the existence of possible antagonistic effects between different gerotherapeutics. Importantly, these challenges may be overcome because evolutionarily conserved molecular signatures of longevity between humans and animals have been identified (unpublished) and novel aging biomarkers that distinguish specific signatures of longevity are emerging [107]. Current knowledge also highlights careful consideration of the combination of geroprotectors that potentially may not lead to synergistic effects, with the example of the known pro-longevity drug metformin [108]. Additionally, COVID-19 research was mentioned in regard to the study of aging as an opportunity to advance geroscience. Aubrey de Grey from SENS Research Foundation, USA, also highlighted this topic and underlined the importance of thinking about COVID-19 in a broader way to target not only the immune system but aging in general, which raises a challenge to disseminate this knowledge to the public to raise awareness of the biology of aging and the possibility of interventions. Hence, novel strategies need to be implemented in order to engage the public into the field of aging.

Another challenging topic discussed by Brian Kennedy, National University of Singapore, Singapore, related to the pros and cons of different ways of testing longevity interventions. Aging interventions would benefit most by applying prevention-based approaches and biomarker discovery [109]. Understanding how different physiological measures and aging clocks correspond to each other could allow targeted testing of different types of aging interventions, including the recently published life-extending molecule 2-oxoglutarate [110]. Further, João Pedro de Magalhães, University of Liverpool, UK, touched upon the topic of longevity interventions and highlighted the exponential growth of pharmacological approaches (DrugAge database), while research in genes associated with longevity have plateaued in recent years (GenAge database). Such shift towards drug discovery is accompanied by the appearance of an anti-aging biotech sector that could bring huge economic benefits in the future [111]. However, the lifespan of anti-aging companies is relatively small due to limitations in the time and ability to validate interventions, likely related to a lack of reliable aging biomarkers. Hence, *in silico*-based approaches are being applied to overcome such limitations to identify either novel genes associated with aging phenotypes [112] or discover drug candidates for life extension [113].

## AI in aging and longevity

At the meeting several talks have been presented showing the power of AI in healthcare and the longevity industry. Kai-Fu Lee, Sinovation Ventures and Sinovation AI Institute, China explained different aspects of artificial intelligence and underlined deep learning to possess amazing attributes and provide great opportunities for the longevity sector. Particularly, deep learning is emerging in every aspect of healthcare and could advance longevity research with new analyses of omics-based big data. Different types of deep learning, including reinforcement learning and transfer learning, were highlighted in the context of building a knowledge-based network for health status prediction. Machine learning techniques are also used to develop a broad range of aging clocks that can be pooled together to conduct AgeMetric, a master predictor of the heath state of an individual [114]. Application of AgeMetric scores were further discussed by Alex Zhavoronkov, Insilico Medicine, Hong Kong who presented how AI-based drug discovery can lead to commercial innovation in longevity. In particular, next generation AI accelerates multiple aspects of biotechnology, including identification of novel targets, a rapid development of small molecules based on known targets [115] and prediction of outcomes of clinical trials. AI outperforms in many aspects humans if enough data is provided. By using different types of data and training a network on relatively healthy people, it is possible to re-train the network on particular disease and identify features that are specific to the diseases. Application of Agemetric scores and other AI-powered preventive medicine strategies in longevity medicine not only enables the prediction of biological age and tracking of healthy state, but also can be used to understand whether an individual is aging faster and how to intervene and slow down aging.

The power of big data was also underlined by Wei-Wu He, Human Longevity Inc., USA, who discussed using big data to personalize aging interventions. These included not only omics data but also whole-body imaging techniques such as MRI. The possibility of combining very large amount of data is a unique opportunity for tailor made longevity solutions for every individual [116].

Sergey Young, Longevity Vision Fund, USA, further unravelled key driving forces within the longevity sector and explained why it is a favorable time to invest in the industry. This includes the appearance of several breakthrough innovations and a growing number of elderly people, along with an exponential increase in the prevalence of chronic age-related disease and unhealthy lifestyle. Further, the integration of AI and technology into medicine, healthcare and the longevity space will likely lead to business opportunities. All this is accompanied by an increase in capital and favourable support in the context of policies and regulations by the government.

Last, Jim Mellon, Juvenescence, UK talked about how Juvenescence focuses on ways for improving human healthspan with the mission to extend healthy lifespan by 8-10 years in the upcoming future. It was highlighted that the biotech world possesses high risks with further complications in predictions of outcomes and achievements. One of the strategies to overcome such limitations is investment in multiple higher risk projects, with the likelihood that one or two of them will succeed. An important aspect of funded projects is the application of AI to accelerate drug development and bring drugs to market as quickly as possible. A Juvenescence pipeline includes focusing on diseases that have a major commercial impact on pro-longevity effects later on, with the example of targeting chronic kidney disease, Alzheimer disease and liver disease. In addition, a fruitful partnership with leading experts in aging and pharma companies facilitates upcoming fully developed products and FDA-approved clinical trials.

## CONCLUSIONS

Current knowledge shows that aging is a very complex but plastic process. Conserved molecular pathways underlining aging can be manipulated using genetic, pharmacological and non-pharmacological approaches to significantly improve the healthspan and lifespan in model organisms, and perhaps humans. A collaborative effort between academic research with a growing number of emerging biotech companies, as well as increased investment funds to accelerate discoveries, will most likely bring effective aging pharmaceuticals in the near future. Undoubtedly, we will see more in the coming ARDD meeting in September 2021 in New York City. The future is bright.
